# Direct and indirect linguistic measures of common ground in dialogue studies involving a matching task: A systematic review

**DOI:** 10.3758/s13423-023-02359-2

**Published:** 2023-08-15

**Authors:** Vincent Bovet, Dominique Knutsen, Marion Fossard

**Affiliations:** 1 Faculté des Lettres et des Sciences Humaines, Institut des Sciences logopédiques, University of Neuchâtel, CH-2000 Neuchâtel, Switzerland; 2 UMR 9193, CNRS, SCALab - Sciences Cognitives et Sciences Affectives, University of Lille, F-59000 Lille, France

**Keywords:** Dialogue, Common ground, Language production, Collaborative approach, Matching task

## Abstract

During dialogue, speakers attempt to adapt messages to their addressee appropriately by taking into consideration their common ground (i.e., all the information mutually known by the conversational partners) to ensure successful communication. Knowing and remembering what information is part of the common ground shared with a given partner and using it during dialogue are crucial skills for social interaction. It is therefore important to better understand how we can measure the use of common ground and to identify the potential associated psychological processes. In this context, a systematic review of the literature was performed to list the linguistic measures of common ground found in dialogue studies involving a matching task and to explore any evidence of cognitive and social mechanisms underlying common ground use in this specific experimental setting, particularly in normal aging and in neuropsychological studies. Out of the 23 articles included in this review, we found seven different linguistic measures of common ground that were classified as either a direct measure of common ground (i.e., measures directly performed on the referential content) or an indirect measure of common ground (i.e., measures assessing the general form of the discourse). This review supports the idea that both types of measures should systematically be used while assessing common ground because they may reflect different concepts underpinned by distinct psychological processes. Given the lack of evidence for the implication of other cognitive and social functions in common ground use in studies involving matching tasks, future research is warranted, particularly in the clinical field.

## Introduction

Dialogue is an extremely frequent activity in daily living and can be defined as interaction involving at least two interlocutors using language in a collaborative manner to reach a common goal (e.g., planning a meeting with a friend) (Clark, 1996). One of the central ideas of such a collaborative approach to dialogue is that interlocutors attempt to reach mutual understanding throughout the interaction in a participatory way; that is, each partner puts a certain amount of individual effort into the dialogue for the current purpose and to ensure successful communication (Allwood et al., 2000; Clark & Wilkes-Gibbs, 1986). Speakers follow *the principle of least collaborative effort*, which involves trying to minimize the total amount of effort put into the dialogue by both partners (i.e., the sum of the individual efforts produced in the interaction) to reach mutual comprehension. In some cases, this involves the speaker putting extra individual effort into message planning in order to facilitate their partner’s comprehension (Clark & Brennan, 1991; Clark & Schaefer, 1989; Clark & Wilkes-Gibbs, 1986; Schober, 1995). In other words, the speakers try to increase the efficacy of the conversation and to reduce the collaborative effort by producing messages that are designed and adapted to their addressee, even if doing so involves increased individual effort, a mechanism called *audience design* (Clark & Murphy, 1982; Clark & Wilkes-Gibbs, 1986; Fussell & Krauss, 1989; Gann & Barr, 2014; Harris et al., 1980; Nückles et al., 2006; Turner & Knutsen, 2021). Consider these two messages during a conversation between friends planning a meeting:

A. “I’m going to a vegetarian Indian restaurant in the old town with a friend whom I met in New York, his name is Mark.”
B. “I’m going to our favorite restaurant with my best friend.”

Suppose that the addressee is a naïve partner who knows neither what “our favorite restaurant” nor “my best friend” refer to. Following the mechanism of audience design, the speaker should design the utterance A to give their partner enough information to correctly understand the message (even though utterance A is lengthier and hence potentially involves higher production costs than utterance B). On the other hand, if the addressee is familiar with what “our favorite restaurant” and “my best friend” mean, the shortened utterance B would be more appropriate to reach mutual understanding efficiently. To adapt their messages according to their conversational partners, interlocutors rely on *common ground*, which is all the information mutually known by the partners, meaning that speakers design utterances for their audience by taking into consideration the knowledge they believe they share with their addressees (Clark & Marshall, 1978, 1981). Part of the common ground is built as the conversation unfolds, implying that each conversational partner’s contributions to dialogue are integrated to their common ground (Clark & Wilkes-Gibbs, 1986). Let us reconsider the example given above. If there is a lack of common ground between the partners (e.g., because the person speaking knows that they have never spoken about this restaurant with their partner and that the latter does not know their best friend), the speaker will tend to favor a longer message with more descriptions, such as utterance A. On the other hand, if “our favorite restaurant” and “my best friend” are mutually known to both partners (meaning they are part of their common ground, for instance because they have already talked about Mark and this specific restaurant in previous conversations), then the speaker should favor more concise and precise references, such as utterance B, to avoid longer and overly specific messages. This example illustrates how the use of common ground leads people to favor the production of references known to their current partner (“my best friend” vs. “a friend whom I met in New York, his name is Mark”), and improves communication efficiency. Thus, the ability to determine and remember what constitutes common ground with a given partner and to access it while speaking is crucial for good communication in everyday life.

In this review, we are interested in how the construction and use of common ground may be measured in experimental settings. One way of assessing common ground from the addressee’s perspective is by using eye-tracking apparatus that enables us to capture the matcher’s eye movements and directions during a referential communication task (Epley et al., 2004; Keysar et al., 2000; Metzing & Brennan, 2003; Wu et al., 2013). For example, in Metzing and Brennan’s (2003) study, a participant interacted with a confederate speaker who repeatedly referred to objects using the same references (e.g., “the shiny cylinder”), leading to the construction of a *conceptual pact* (i.e., a temporary agreement regarding how to refer to a given referent; such pacts belong to the partners’ common ground; Brennan & Clark, 1996) for each item. Then, the original or a new confederate referred to objects with the original or a new reference (e.g., “the silver pipe”) (Metzing & Brennan, 2003). The results showed that participants were slower to look at the target item when a new reference was used, particularly in the presence of the original confederate, because they expected the confederate speaker to use their common ground (“the shiny cylinder”) instead of a new reference (“the silver pipe”). The analysis of the matcher’s eye movement was interpreted here as a measure of how common ground is used from the addressee’s perspective.

The construction and use of common ground can also be assessed from the speaker’s perspective. One of the most frequent and influential way of doing so is to use referential communication tasks, which often involve the presentation of abstract tangram figures to participants – although this is not systematically the case (Clark & Wilkes-Gibbs, 1986; Horton & Gerrig, 2002; Isaacs & Clark, 1987; Knutsen et al., 2019; Krauss & Weinheimer, 1966; Schober & Clark, 1989). In such a paradigm, a participant (the director) has to refer to tangrams to help their partner (the matcher) to arrange them in a correct order. The same task is repeated across several trials with the same tangrams placed in a different order. The use of tangrams is particularly relevant in studying common ground construction and use, as dialogue partners are usually unfamiliar with these shapes (i.e., they have no common ground regarding how to refer to them). During the first trial, the participants must build this common ground by reaching an agreement as how to refer to the tangrams. This usually involves a fair amount of negotiation, reflected by the number of words and speech turns necessary to complete the task. During the second trial (and all subsequent trials) there is no need for such negotiation, as the partners already share common ground and can use it to perform the task more efficiently. Thus, studying the way in which dialogue partners refer to the same tangrams over trials enables us to describe common ground construction (during the first trial) and subsequent use (in subsequent trials).^1^ One result that has been well described is that interlocutors tend to reduce the number of words and speaking turns across the trials, increasing communication efficiency (Clark & Wilkes-Gibbs, 1986). They use longer descriptions in the initial trial such as *“looks like a person who’s ice skating, except they’re sticking two arms out in front,”* while simplifying and optimizing their utterances as they refer to the same tangrams repeatedly to finally use a short reference such as *“the ice skater”* in the last trial. This phenomenon reflects how interlocutors come to agreement on specific conceptual pacts while building common ground, and how they use the information they know they share with their addressee to produce shorter and clearer messages. Across the trials, speakers also tend to use more definite articles (*“the ice skater”*) instead of indefinite references (*“an ice skater”*) to mark that they believe that their partner can identify uniquely the target among other potential referents and therefore that the reference is mutually known (i.e., part of their common ground). Thus, the reduction in the number of speaking turns and words and the increase of definite articles across trials are interpreted as reflecting the way in which the construction of common ground (in trial one) or the use of this common ground (in subsequent trials) influences the content of the participants’ utterances.

It is interesting to notice that both kinds of study rely on different measures to assess common ground construction and use. On the one hand, studies assessing common ground from the addressee’s perspective mainly use receptive, non-strictly linguistic measures such as eye movements to determine whether participants are resorting to their common ground to interpret references. On the other hand, studies assessing common ground from the speaker’s perspective mainly use linguistic measures such as reference content or number of words produced to determine whether participants are resorting to their common ground during speech production. In the current study, we decided to focus specifically on linguistic measures found in studies using a matching task (i.e., a collaborative experiment where a participant must help their partner to identify different elements in a specific order on several trials using language). Although it would have been interesting and relevant to also include non-linguistic measures in this review, we believe that starting by focusing only on linguistic measures is justified by the lack of consensus in the literature regarding which exact construct is supposed to be measured by each linguistic measure of common ground. For instance, the decrease in the number of words is often taken to be an indicator of the presence of common ground, but it can also be interpreted as reflecting the decrease in the amount of collaborative effort produced by the dyad (in this case, the number of words produced by each participant is interpreted as reflecting the effort produced by each participant). A decrease in the number of words during the interaction is also supposed to reflect people engaging in audience design. There is therefore an urgent need to better understand what the different measures are and which constructs they reflect. Interestingly, whereas some linguistic measures of common ground work at a more macro level of discourse, acting as “discourse-shaping indicators” and reflecting the form of the discourse as a whole (e.g., the number of words per utterance), others are more directly related to the referential content of the discourse, acting as “reference markers” (e.g., which reference is used, whether it is definite or indefinite). In order to determine whether this distinction is relevant, and whether it can help us better understand how common ground is assessed in experimental studies, the main goal of this review is to list the linguistic measures used in the relevant studies that assess common ground construction and subsequent use by applying the distinction between discourse-shaping indicators and reference markers. Our choice to focus only on the matching task (and not on other paradigms that have been used to study common ground construction and use) is mainly motivated by the fact that this paradigm is used in a very high number of dialogue research studies. What is more, although comparing the results obtained using different research paradigms would have been interesting, comparing the use of different linguistics measures of common ground in studies that used the same methodology will help us shed light on the lack of consensus regarding the constructs these measures are supposed to reflect.

Beyond the first main objective, we are also interested in investigating the potential cognitive mechanisms related to and underlying the construction and use of common ground, particularly the memory processes, in studies among neuropsychological and aging populations. The link between memory processes and the use of common ground was already established by Clark and Marshall in 1981 when they proposed that while interacting together, interlocutors encode the information exchanged in the conversation jointly with the memory of the presence of both their partner and themselves (notion of triple co-presence). To determine whether information is mutual or not for designing their utterances, partners try to recall the memory of this triple co-presence (the target-information, their partner and themselves) which is stored in memory. According to Clark and Marshall (1981), common ground is represented in specialized memory structures for dialogue that encode exclusively the information related to a specific partner.

However, Horton and Gerrig (2002, 2005a, 2016) stipulated in their memory-based approach that access to common ground relies on more “ordinary” episodic memory processes, meaning that those memory mechanisms are not specific to dialogue. According to this approach, an association between the information exchanged and the partner is encoded automatically and incidentally as the conversation progresses. Then, each conversational partner serves as a contextual cue for the other to retrieve all the information previously exchanged and therefore access the common ground. This memory process is called *resonance*: the presence of a cue (the partner) in the working memory can activate all the information associated with this cue in the long-term memory. Thereby, access to common ground can be considered an automatic and low-cost cognitive process. Given the involvement of memory systems in the use of common ground according to the theoretical models, it seems relevant to study common ground by also examining the participants’ conversational memory – that is, memory for conversation content (what was said) and source (who said what to whom) (Fischer et al., 2015; Keenan et al., 1977; Knutsen & Le Bigot, 2014; MacWhinney et al., 1982; Stafford & Daly, 1984). The present research also aims to report whether the studies of interest investigated these aspects.

Apart from the memory processes, other cognitive functions could potentially be involved in the construction and use of common ground. Firstly, for some authors, designing utterances and using common ground in dialogue require intact abilities in cognitive and affective theory of mind (Achim et al., 2015; Moreau et al., 2015, 2016), meaning that being able to access others’ mental states such as their knowledge, thoughts, feelings, or emotions could help the conversational partners to assess whether a particular piece of information is mutually known or not, and to support them in the referencing process (e.g., because I am able to represent my partner’s knowledge and thoughts, I am able to use this representation to determine whether my partner is likely to know who my best friend is).

Secondly, the implication of high executive control in language processing has been well described (for a review, see Ye & Zhou, 2009), particularly in perspective-taking in comprehension in conversational settings (Brown-Schmidt, 2009; Lin et al., 2010; Wardlow, 2013). It could be hypothesized that using common ground involves executive control processes such as inhibition, shifting, and updating, the three elementary executive components described by Miyake et al. (2000): while interacting, the interlocutors must *inhibit* all the potential competitors to select the most partner-appropriate reference for a given concept (e.g., “the friend I met in New York”, “my best friend”, “Mark”, “Lily’s brother”, etc.), to take into consideration the information of the context and the knowledge shared with their partner *flexibly* and to constantly *update* the information integrating the common ground as the conversation progresses. We are interested here in listing any statistical evidence of a link between the linguistic measures of common ground and any scores in experimental tasks evaluating theory of mind and executive control processes. In this respect, developmental research such as aging and neuropsychological studies could be useful and very informative to determine the implication of these cognitive functions in using common ground and to better describe any difficulty observed in dialogue in specific populations. Thereby, we are also interested in answering whether there are studies that assess common ground using a matching task with healthy aged participants or patients with neurological or psychiatric affections.

To sum up, the main goal of this review is to answer the following research questions:

1. What are the different linguistic measures of common ground construction and subsequent use examined in dialogue settings using a matching task? To answer this question, we will attempt to apply our distinction between measures that represent indicators of discourse-shaping and measures that are reference markers.
2. Are there differences in the experimental settings (stimuli, role in the conversation, nature of the partner) in the relevant studies?
3. Are there any studies using a matching task that assess conversational memory in terms of content and source, and what is known about their implication in the construction and the use of common ground?
4. Is there any evidence of a link between common ground and other social and cognitive functions (executive functions, theory of mind)?
5. What are the different types of population in terms of age or neurological/psychiatric disorders? Is there a different result pattern depending on the measures used (discourse-shaping indicators and/or reference markers) in those populations?

## Method

### Information sources, search strategy, and eligibility criteria

Three electronic databases were consulted for this review: Scopus, Psyc’INFO, and Pubmed. The Scopus and Psyc’INFO databases were used because of the large number of available publications they include and because they list studies on dialogue, and Pubmed was used to search for possible studies on patients with neurological or psychiatric pathology. We limited our selection to studies published in English scientific journals with no time limitation. The keywords used in the search included terms associated with the concept of common ground and terms reflecting dialogue. We adapted the Boolean operators and truncators to the specificities of each database: “common ground” OR “audience design” OR “lexical entrainment” OR “joint effort*” OR “shared information*” OR “referencing process” AND conversation OR “communication partner*” OR “collaborative interaction*” OR “collaborative dialogue” OR “referential communication” OR “language production” OR “language comprehension”^2^ OR “matching task.” This review was conducted according to the Preferred Reporting Items for Systematic Reviews and Meta-Analyses (PRISMA) guidelines (Page et al., 2021). To assess the research questions, the eligibility criteria were set as follows:

*Inclusion criteria*

1. Studies that included experimental design with an oral and interactive matching task between at least two adult participants. The paradigm should allow the partners to engage in spontaneous dialogue and the instructions should explicitly mention that they could talk to each other freely.
2. Studies that included linguistic measures of the construction and the use of common ground. Studies that focus on common ground use, but not on how this common ground was built in the first place, were thus not included, as specified below.
3. Studies that included healthy adult populations or those with neurological and/or psychiatric disorders.

*Exclusion criteria*

1. Studies that were published in books or in conference papers.
2. Studies that included interaction between adult and children/teenagers or with electronic devices (e.g., computers).
3. Studies that included non-linguistic measures of common ground (such as eye-tracking studies). One study that involved both linguistic and non-linguistic measures of common ground was included in our work with a focus on linguistic measures.
4. Studies that only measured the use of common ground and not its construction during a dialogue. This exclusion criterion led us to discard studies that manipulated the information shared between the partners (privileged vs. common ground) before a conversational task in which a director had to help their partner identify a target among distractors (e.g., Heller et al., 2012).

### Study selection

**Fig. 1:**
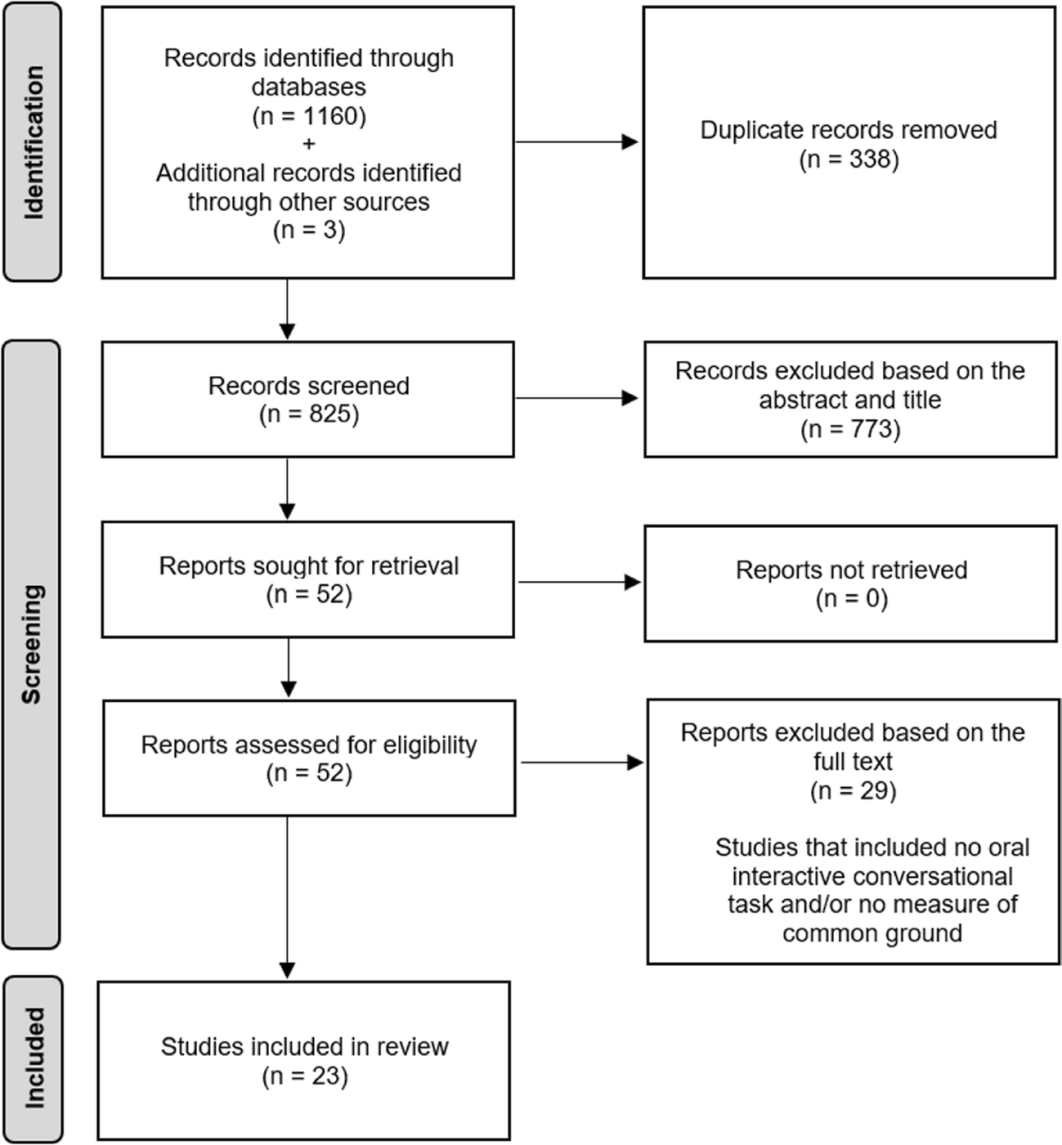
Research process and number of studies included in the review

Database searches were performed in October 2021 and were updated in September 2022. The first selection step was performed independently by the first author and seven other examiners (Master’s degree students) paired in three different groups. This first step was based on the titles and abstracts of each record yielded by the literature search after all duplicates had been removed by the first author. According to the eligibility criteria presented above, irrelevant studies were excluded. Then, after the first selection step, the same investigators read the full text of the remaining articles. For the latter, the same eligibility criteria were used for the inclusion/exclusion of the articles. During both steps of the selection process, any disagreements were resolved by discussion to find consensus. Out of the 825 studies initially retained, 23 publications finally met the eligibility criteria (Fig. 1). It should be noted that three studies (Lysander & Horton, 2012; Moreau et al., 2015, 2016), which did not emerge from the research in the databases because they did not include any of our keywords about common ground but which met the eligibility criteria, were added manually.

### Data extraction and analysis

For each selected study, the following relevant data were extracted independently by the same investigators using a data extraction table. Firstly, we identified the different types of measures of common ground used in the relevant studies and then we applied the classification, i.e., the two different types of linguistic measures of common ground proposed in the *Introduction*. When the measure was directly related to the referential content of the discourse (i.e., derived from the analysis of the type of articles or the lexicon used), it was classified as a reference marker. On the other hand, when the measure took root at a more macro level of discourse, reflecting the shape of the discourse as a whole, it was classified as a discourse-shaping indicator.

Then, we listed the characteristics of the populations (age, gender, normal healthy adults vs. clinical population) and the experimental design such as the type of the matching task, whether visual contact was allowed or not, the type of role in the conversation (director-matcher or alternated role), and the type of partner (experimenter, unknown, or relative).

Finally, we looked for any statistical links between common ground measures and other social and cognitive functions (episodic memory, conversational memory in terms of content and source, executive functions, theory of mind, personality traits). The data were then analyzed using descriptive tables to support comparisons between the studies and to answer the research questions.

## Results

The data extracted from the 23 studies included in this review are summarized in Table 1 (publications that included only healthy participants) and in Table 2 (publications that included clinical populations).

**Table 1:** Characteristics of the publications retained after selection using exclusion/inclusion criteria including only healthy participants

Study	Target group	Conversational task			CG measure	Link with cognitive functions
		Type of task	Type of role	Type of partner		
Bangerter et al., (2020)	176 University students	Matching tangrams	Director	Unknown	Mixed (1, 3, 5)	No
Bortfeld & Brennan (1997)	60 University students	Matching pictures	Mixed	Unknown	Mixed (1, 4)	No
Brennan & Clark (1996)	72 University students	Matching pictures	Mixed	Unknown	Mixed (4, 7)	No
Clark & Wilkes-Gibbs (1986)	16 University students	Matching tangrams	Director	Relative or unknown	Mixed (1, 2, 5)	No
Horton & Gerrig (2005b)	72 University students	Matching pictures	Director	Relative or unknown	Mixed (5, 7)	No
Horton & Spieler (2007)	24 aged adults (M=72.7)	Matching pictures*	Mixed	Unknown	Mixed (1, 2, 4)	No
Hupet et al. (1993)	20 aged adults (M=70)	Matching tangrams	Mixed	Relative or unknown	Mixed (1-5)	No
Liu et al. (2021)	96 University students	Matching art installations	Director	Relative or unknown	Reference markers (5)	No
Lysander & Horton (2012)	16 aged adults (M=76.6)	Matching tangrams and faces	Mixed	Unknown	Discourse shaping (2)	No
McKinley et al. (2017)	72 University students	Matching pictures	Mixed	Relative or unknown	Discourse shaping (1)	Memory of content and source
Wilkes-Gibbs & Clark (1992)	96 University students	Matching tangrams	Director	Relative or unknown	Mixed (1, 3, 6)	No
Yoon & Brown-Schmidt (2014)	96 University students	Matching tangrams*	Director	Unknown	Discourse shaping (1, 7)	No
Yoon & Brown-Schmidt (2018)	120 University students	Matching tangrams*	Director	Relative or unknown	Mixed (1, 5, 7)	No
Yoon & Brown-Schmidt (2019)	84 University students	Matching tangrams*	Director	Unknown	Mixed (1, 5, 7)	No

CG measures: 1. Number of words. 2. Number of speaking turns. 3. Definite/indefinite references. 4. Reuse of references. 5. Conceptualization. 6. Time. 7. Dysfluencies-hedges. *Only the data from the training phase (matching task) were extracted and analyzed

**Table 2:** Characteristics of the publications retained after selection using exclusion/inclusion criteria including only clinical populations.

Study	Target group	Conversational task			CG measure	Link with cognitive functions
		Type of task	Type of role	Type of partner		
Duff et al. (2006)	4 patients with bilateral hippocampal damage	Matching tangrams	Director	Relative	Discourse shaping (1, 6)	No
Duff et al. (2011)	6 patients with bilateral hippocampal damage	Matching tangrams	Director	Relative	Reference markers (3)	No
Feyereisen et al. (2007)	13 patients with AD	Matching tangrams	Director	Experimenter	Mixed (1-5)	No
Gupta et al. (2011)	5 patients with bilateral amygdala damage	Matching tangrams	Director	Relative	Discourse shaping (1, 6)	No
Gupta et al. (2012)	7 patients with vmPFC damage	Matching tangrams	Director	Relative	Mixed (1, 3, 6)	No
Moreau et al. (2015)	20 patients with MCI	Matching tangrams	Director	Experimenter	Mixed (1, 2, 3)	No
Moreau et al. (2016)	20 patients with AD	Matching tangrams	Director	Experimenter	Mixed (1, 2, 3)	No correlation with episodic memory and executive functions
Nadig et al. (2015)	13 patients with ASD	Matching tangrams	Director	Experimenter	Mixed (2, 4-6)	No
Yoon et al. (2017)	4 patients with bilateral hippocampal damage	Matching tangrams	Director	Experimenter	Mixed (1, 3)	No

*CG measure: 1. Number of words. 2. Number of speaking turns. 3. Definite/indefinite references. 4. Reuse of references. 5. Conceptualization. 6. Time. 7. Dysfluencies-hedges. AD, Alzheimer’s disease; vmPFC, ventromedial prefrontal cortex; MCI, mild cognitive impairment; ASD, Autism Spectrum Disorder*

1. *What are the different linguistic measures of common ground construction and subsequent use examined in dialogue settings?*

Of the 23 publications included, we listed seven different linguistic measures used to assess common ground in experimental settings using a matching task and ranked them in order of frequency, as shown in Table 3. We also applied the distinction between measures that represent indicators of discourse-shaping and measures that are reference markers for each measure of common ground found in the literature. According to this distinction, we listed four measures that represent indicators of discourse-shaping: the number of words produced during the dialogue task (by the director or the dyad calculated for the initial description, per item or per trial), the number of speaking turns between the interlocutors (calculated per item or per trial), the dysfluencies – hedges (that included the planning time before producing the first utterance, the use of try markers, and the lengthening on the article),^3^ and the time taken to accomplish the task.

**Table 3:** List of the linguistic measures of common ground found in the selected articles

Linguistic measures of CG	Type of measure	Frequency
Number of words	Discourse shaping	17
Ratio of definite/indefinite references	Reference markers	9
Conceptualization	Reference markers	9
Number of speaking turns	Discourse shaping	8
Reference reuse	Reference markers	6
Dysfluencies – hedges	Discourse shaping	5
Time	Discourse shaping	5

*The frequency corresponds to the number of times a given measure is found in the selected studies*

We listed three different measures associated with reference marker production. These measures were considered reference markers if they derived directly from the analysis of the type of references produced such as the articles and the lexicon used by the speaker. Nine studies (three based on healthy participants and six based on clinical populations) used the ratio of definite/indefinite references calculated out of the total number of words produced by the speaker to assess common ground (Bangerter et al., 2020; Duff et al., 2011; Feyereisen et al., 2007; Gupta et al., 2012; Hupet et al., 1993; Moreau et al., 2015, 2016; Wilkes-Gibbs & Clark, 1992; Yoon et al., 2017). We also listed nine studies (seven based on healthy participants and two based on clinical populations) that used markers of conceptualization such as the number of labels produced by the speaker (i.e., the use of a very short noun phrase without elaboration that tends to increase across the trials) and markers of reconceptualization such as the number of descriptive words or the number of new content words produced (that tend to decrease across trials) (Bangerter et al., 2020; Clark & Wilkes-Gibbs, 1986; Feyereisen et al., 2007; Horton & Gerrig, 2005b; Hupet et al., 1993; Liu et al., 2021; Nadig et al., 2015; Yoon & Brown-Schmidt, 2018, 2019). Unlike the measure of the number of total words produced that was classified as a discourse-shaping indicator, the markers of conceptualization and reconceptualization were considered reference markers because they derived directly from the analysis of the way in which the participants referred to the pictures during the task. Markers of reconceptualization were particularly used to assess the speaker’s potential adjustments in experimental settings where a new naïve matcher joins the current task. These markers reflect how interlocutors agree and converge on specific conceptual pacts while building common ground, and how speakers tend to use this information to produce a clearer message. Finally, six studies (four based on healthy participants and two based on clinical populations) considered the reuse of previously produced references to assess common ground. In order to do this, two studies analyzed how the speaker took into account and reused the descriptions initially produced by the matcher during the following trials (Horton & Spieler, 2007; Nadig et al., 2015). In four other studies, a reference was categorized as reused if the same initially produced expression was reproduced in the next trials (Brennan & Clark, 1996; Feyereisen et al., 2007; Hupet et al., 1993). Bortfeld and Brennan (1997) also included the reference reuse as a measure of common ground and detailed the six different categories they applied for each expression produced more precisely: (A) verbatim equivalence (the exact same expression is reproduced), (B) propositional equivalence (the exact same content words produced in a different order), (C) equivalence in content words but shorter expression (with no more than one less modifier), (D) equivalence in content words except one is different, (E) some content words are the same but there is no criteria for a category between A and D, (F) a totally new expression is produced. Overall, these studies showed an increase in the frequency of reuse of the same references as the common ground is established.

Of the 23 publications, five studies (three based on healthy participants and two based on clinical populations) included only discourse-shaping indicators, two studies (one based on healthy participants and one based on a clinical population) included only reference markers, and 16 studies (ten based on healthy participants and six based on clinical populations) included mixed measures (discourse-shaping indicators and reference markers).

2. *Are there differences in the experimental settings (stimuli, role in the conversation, nature of the partner) in the relevant studies?*

The majority of the studies used tangrams, even though few studies used other abstract images or pictures. The studies differ in the number of items selected and in the number of trials. Note that four studies (all based on healthy participants) interested particularly in multiparty conversation used a slightly different experimental paradigm (Horton & Spieler, 2007; Yoon & Brown-Schmidt, 2014, 2018, 2019). In this paradigm, firstly two participants took part in the matching task with tangrams/pictures to get familiar with the items. Then, the participants engaged in an identification phase where the director was required to refer to a target (a familiar tangram/picture or a new one) between three distractors to help the matcher to identify the correct one. At this stage, a third naïve matcher joined the conversation. The authors were mainly interested in how the use of common ground from the director’s perspective is affected by the presence of different listeners. Given our selection criteria, we only extracted and analyzed data from the training phase. Results from Horton and Spieler (2007) are discussed below with the question of normal aging. Yoon and Brown-Schmidt (2014, 2018, 2019) used the number of words, the dysfluencies-hedges (latency before the utterance, use of try markers, lengthening on the definite article), and the reconceptualization (use of different descriptive words across trials) to assess common ground. These studies illustrated how speakers can distinguish the different common ground they share with different partners, and how they tend to adapt utterances to the least-knowledgeable partner when they engage in multiparty conversations. Overall, the common ground measures showed a pattern of results similar to that obtained in the standard matching tasks.

One study (based on healthy participants) used a more ecological approach by going outside of the laboratory to assess common ground. Liu et al. (2021) proposed a dialogue task in a naturalistic setting where participants had to find public art installations in downtown Santa Cruz. Even though it may seem surprising to include this study given that the experiment took place in a real-world environment, we believe that the experimental paradigm used can be considered as a matching task: a participant located in a campus lab had to help their partner, situated in the city center, to identify five targets (art installations) across two trials using a cellphone. Authors used a measure of conceptualization (i.e., the number of descriptions produced by the director for each target in each trial) to assess common ground across the trials. This task shows a pattern of results similar to those obtained in the more experimentally controlled tasks: the number of descriptions produced by the director tend to decrease in the second trial, reflecting the conceptualization process.

We were also interested in other characteristics of the experimental setting such as the type of role in the conversation and the nature of the conversational partner. In 17 studies (eight based on healthy participants and nine based on clinical populations) the director was always the same participant, while in six studies (all based on healthy participants) the participants alternated between the roles of director and matcher. In 18 studies (14 based on healthy participants and four based on clinical populations), the participant carried out the conversational task with either a relative or an acquaintance, while in five clinical studies the conversational partner was an experimenter playing the role of the matcher.

3. *Are there any studies that assess conversational memory in terms of content and source, and what is known about their implications in the construction and the use of common ground?*

Of the 23 publications included, only one study (based on healthy participants) performed measures of both conversational memory and common ground, and was mainly interested in how the construction of common ground during the dialogue affected the memory of the content and the source (McKinley et al., 2017). In this study, each participant performed a matching task with two different partners (four rounds in total, each composed of three trials with the same image and the same partner). After the dialogue phase, participants took part in a recognition test where they saw old and new pictures. For each item, they had to make judgments about the content (they were asked to answer “yes” if they thought they had already seen the picture) and the source (they were asked to answer with which partner they saw the picture). The authors were particularly interested in how forming common ground can affect conversational memory. They calculated a “quality index” of the development of common ground across trials by making the difference between the number of words produced in trial one versus that of trial three. A higher score indicated better conceptualization and thus a more solid construction of common ground. The results showed that this index was a significant predictor of both scores obtained in the recognition test, meaning that forming common ground tends to promote conversational memory for both content and source.

It should be noted that one study showed no correlation between scores obtained in a task assessing verbal episodic memory and linguistic measures of common ground in patients with Alzheimer’s disease (AD) (Moreau et al., 2016).

4. *Is there any evidence of a link between common ground and other social and cognitive functions (executive functions, theory of mind)?*

Of the 23 publications included, there is only one study that investigated whether there was a statistical link between executive functions and common ground measures (Moreau et al., 2016). No significant correlation was found between scores obtained in tasks assessing executive functions and linguistic measures of common ground (number of words and speaking turns, ratio of definite/indefinite articles) in patients with AD. Moreover, there have been no studies to test for a potential link between linguistic measures of common ground and standardized tests assessing theory of mind.

5. *What are the different types of population in terms of age or neurological/psychiatric disorders? Is there a different result pattern depending on the measures used (discourse-shaping indicators and/or reference makers) in those populations?*

Of the 23 publications included, 14 studies involved healthy participants and nine studies focused on clinical populations. Among the publications involving healthy participants, three studies were specifically interested in normal aging, and showed no significant difference in the construction and use of common ground between younger and older healthy adults (around 70 years old) in a matching task except that aged participants initially generally needed more words and more speaking turns to accomplish the task (Horton & Spieler, 2007; Hupet et al., 1993; Lysander & Horton, 2012).

Among the research in the clinical field, three studies focused on patients with bilateral hippocampal brain lesions (Duff et al., 2006, 2011; Yoon et al., 2017), two on patients with AD (Feyereisen et al., 2007; Moreau et al., 2016), one on patients with mild cognitive impairment (MCI) (Moreau et al., 2015), one on a patient with bilateral amygdala brain lesions (Gupta et al., 2011), one on patients with bilateral ventromedial prefrontal brain lesions (vmPFC) (Gupta et al., 2012), and one on adults with autism spectrum disorder (ASD) (Nadig et al., 2015).

Several studies on patients with amnesic cognitive profile (bilateral hippocampal brain lesions and AD-MCI patients) showed different result patterns in the use of common ground between controls and clinical groups depending on the type of measures used to assess common ground (Duff et al., 2006, 2011; Moreau et al., 2015, 2016). The amnesic groups only differed from controls on the reference markers (e.g., ratio of definite/indefinite articles), meaning that the increase in use of definite articles across trials was significantly higher for the controls. However, there was no significant difference between patients and controls when discourse-shaping indicators were used to assess common ground (e.g., number of words and speaking turns), meaning that the decrease of those measures across trials was very similar between patients and controls. Nevertheless, such dissociation between both types of measures was not found in other studies where amnesic patients differed from controls in both types of measures (Feyereisen et al., 2007; Yoon et al., 2017).

Three other clinical studies included in this review investigated the social and emotional mechanisms and neural substrates that may be involved in common ground use. Gupta et al. (2011) showed that a patient with bilateral amygdala damage who exhibits deficits in various aspects of basic social and emotional processing also presented difficulties in the use of common ground during a matching task. The decrease in the number of words produced and the time taken to complete the task across trials was significantly less for the patient compared to controls. However, the same authors conducted a similar study with seven patients with vmPFC damage, a brain area known in particular to be involved in theory of mind, and found no significant difference between the clinical group and the controls using both discourse-shaping indicators (number of words and time to complete the task) and reference markers (percentage of definite references) despite patients’ post-morbid changes in their social and emotional functioning (Gupta et al., 2012). Finally, Nadig et al. (2015) studied adult patients with ASD to assess the importance of theory-of-mind abilities in building and using common ground and to investigate the partner-specificity of common ground. To do so, they used a matching task with tangrams where participants played the role of the director across the trials and interacted with either the same experimenter during the whole task or with two different experimenters (i.e., the first experimenter stayed during three trials and then was replaced by a new and “naïve” experimenter for the last two trials, meaning that the participants did not share common ground about the tangrams with the new experimenter). The results showed that patients with ASD did not differ from neurotypical adults in their ability to build common ground with a specific partner on both reference markers (number of descriptions) and discourse-shaping indicators (number of speaking turns and time). On the other hand, ASD patients needed more time than controls to complete the task, particularly for the last two trials in the new-experimenter condition, and were less likely to incorporate new descriptions produced by the new experimenter in their utterances during the last trial. Thus, despite similarities in building common ground, ASD patients seem to require more effort to use the common ground they share with a specific partner adequately, and tend to reuse the same references regardless of their conversational partner in an inflexible manner.

## Discussion

The goal of this systematic review was to list the different linguistic measures of common ground used in matching tasks by applying the distinction between discourse-shaping indicators and reference markers, to explore the different characteristics of the experimental setting used to assess common ground, and to investigate the social and cognitive mechanisms (memory processes, theory of mind, executive functioning) related to and underlying the common ground particularly in neuropsychological and normal aging studies. Twenty-three studies met all selection criteria and were included in the review.

### A conceptual distinction between “direct” and “indirect” measures of common ground

In total, seven different linguistic measures of common ground were listed in this review. Four of them were classified as discourse-shaping indicators (number of words produced, number of speaking turns, dysfluencies-try markers, time), while three of them were classified as reference markers (ratio of definite/indefinite articles, markers of conceptualization and reconceptualization, reference reuse).

Using the distinction between both types of measures when assessing common ground is relevant for several reasons. First, on a conceptual level, we suggest that both types of measures may reflect different concepts proposed by the collaborative approach. We propose that reference markers represent a *direct* measure of common ground use. The measures are performed through the analysis of the references produced such as the type of article or lexicon used. For instance, the reference reuse constitutes an explicit mark of common ground use in the discourse surface, allowing us to access the content of common ground (e.g., “I know that the reference *Mark* is part of the common ground I share with my partner, so I reuse this same reference through the conversation”). What is more, reference markers directly reflect the establishment and use of conceptual pacts, which tallies with the content of the partners’ common ground. Since the measures are directly performed on the referential content of the discourse produced, we believe they assess the use of common ground in a direct manner. On the other hand, discourse-shaping indicators seem to reflect the common ground more *indirectly* by assessing the general form of the discourse. Indeed, the latter measures do not focus on the content of common ground per se; rather, they capture aspects of discourse that are believed to be influenced by the presence of common ground (e.g., we suppose that the number of words produced decreases across trials in the matching task because interlocutors share more and more common ground during the interaction). In short, we argue that the content of common ground can directly be measured by reference markers while discourse-shaping indicators represent a more general measure of the discourse that can be influenced indirectly by the presence of common ground. The relevance of this conceptual distinction is supported by clinical studies, which are discussed below.

### Highlighting the need to assess common ground in more natural contexts

One of the other goals of this review was *to explore the potential differences in experimental characteristics in the matching tasks*. Most of the studies included in this review used the classic matching task with a procedure similar to the one proposed by Clark and Wilkes-Gibbs (1986), differing in the number and the type of the items selected (tangrams, other abstract figures, pictures) and in the number of trials. Research in multiparty conversations used a slightly different experimental task where the participants firstly took part in the classic matching task and then engaged in an identification task with the same conversational partner and a third naive participant (Yoon & Brown-Schmidt, 2014, 2018, 2019). This setting allowed the authors to study how the use of common ground from the director’s perspective was influenced by the presence of different listeners.

There is only one study included in this review that assessed common ground in a more naturalistic setting by going outside the laboratory, such as finding art installations in a city (Liu et al., 2021). Even though this study showed a similar pattern of results to that obtained in studies using the classic matching task, there is a need for a greater variety of ecological tasks in order to corroborate and to generalize to different dialogic contexts the results obtained in more controlled experimental settings such as the matching task with tangrams. This statement is particularly relevant for research in clinical fields since all the studies with a neurological and/or psychiatric population included in this review applied a strict and controlled experimental paradigm by using the matching task with tangrams and by constantly controlling the dialogic role of the patient (i.e., the patient was the director in all the clinical studies), which is not fully representative of a dialogue in everyday life. Moreover, five out of the nine clinical studies required a confederate to play the role of the conversational partner while the biases generated by such a procedure, particularly when the confederate is the addressee, are substantial (for a theoretical review, see Kuhlen & Brennan, 2013). Overall, this review shows that linguistic measures of common ground are predominantly carried out on controlled experimental tasks in a laboratory setting, and highlights the need to assess common ground in more natural contexts too.

#### Evidence for the implication of memory systems in the use of common ground from the clinical field

The relevance of the distinction between both types of measures is supported by the results from neuropsychological studies, which are very informative about the cognitive processes underlying common ground use. As already mentioned above, the implication of memory systems in the construction and the use of common ground have been well described in the theoretical models proposed by Clark and Marshall (1981) and by Horton and Gerrig (2002, 2005a, 2016). In their work with brain-damaged patients, Duff and Brown-Schmidt (2012, 2017) highlighted the contribution of hippocampal brain regions, known to support the encoding and retrieval of episodic memories, in online processes such as language processing. Moreover, several studies on patients with episodic memory impairment (bilateral hippocampal brain lesions and AD-MCI patients) were included in this review. Interestingly, except for two studies that showed significant differences for both types of measures between patients and controls (Feyereisen et al., 2007; Yoon et al., 2017), four other studies showed different result patterns between controls and clinical groups in the use of common ground depending on the type of measures used to assess common ground (Duff et al., 2006, 2011; Moreau et al., 2015, 2016). When discourse-shaping indicators were used to assess common ground use, patients did not differ significantly from controls, that is, the decrease in the number of words produced and of speaking turns was similar across trials for both groups. In contrast, patients differed significantly from controls when reference markers (i.e., ratio of definite/indefinite articles) were used to assess common ground.

Since the patients recruited in these clinical studies are known to be amnesic (i.e., they present a strong impairment of their declarative episodic memory while other memory systems such as procedural memory are preserved) and that they differ from controls depending on the measure used to assess common ground (Duff et al., 2006, 2011; Moreau et al., 2015, 2016), we could assume that both types of measure (direct and indirect) represent different concepts that are underpinned by different memory systems. We could argue that reference markers such as the ratio of definite/indefinite articles are linked to a declarative episodic memory system since the amnesic patients showed significant differences compared with controls on those reference markers (e.g., they used less definite articles than controls). Their difficulties in using definite articles to mark common ground may be linked to their impaired declarative memory. On the other hand, discourse-shaping indicators could be underpinned by an implicit and procedural memory system since these patients showed no difference compared with controls on those markers (e.g., they tended to use less words across trials than the control subjects). Their ability to reduce the number of words and speaking turns across trials despite their declarative memory impairment could be linked to their preserved procedural memory. These results could suggest that reference markers directly assess the content of common ground, meaning the episodic memory of past conversations (e.g., “I remember that we agreed how to refer to this tangram so I reuse the same reference later in the dialogue”), while discourse-shaping indicators constitute an indirect measure of common ground that relies on more automatic and procedural memory system (e.g., “I have implicit knowledge of how I have to communicate in order to reduce the collaborative effort and to design utterances accordingly”). Altogether, results in clinical fields might support the idea that both types of common ground measures may be underpinned by different memory systems.

### Towards a declarative/procedural model of dialogic skills?

This distinction between explicit episodic and implicit procedural memory processes, which may be engaged during dialogue, could echo the declarative/procedural model of lexicon and grammar proposed by Ullman et al. (1997) based on their work in verb inflectional morphology. These authors proposed that the inflection of regular verbs is underpinned by procedural memory, a system of implicit rules that includes mental grammar, while the inflection of regular verbs is supported by declarative memory processes, a system allowing the learning, representation, and recovery of mental lexicon (Ullman, 2004, 2016; Ullman et al., 2005). Although this declarative/procedural model is not directly applicable to dialogue, given the lack of empirical evidence, we could postulate that declarative memory processes support some dialogic skills, such as the ability to encode and retrieve shared information with a given partner, while other dialogic skills are supported by a system of implicit rules about how to effectively communicate, such as the ability to reduce collaborative effort. Future research is warranted to further explore this idea.

### Weak evidence for the implication of other social and cognitive mechanisms in the construction and use of common ground

Apart from memory processes, we aimed to investigate the potential link between the use of common ground and other social and cognitive mechanisms such as theory of mind and executive functioning. Only one study included in this review showed no significant correlation between linguistic measures of common ground and scores obtained in executive tasks in patients with AD (Moreau et al., 2016), while no other study investigated those potential links, highlighting the lack of data analysis in this field.

Developmental research in normal aging does not provide clear evidence for the implication of executive functioning in the use of common ground. Interestingly, despite the decline generally observed in executive skills with normal aging such as inhibition, mental flexibility, and decision making (for reviews, see Harada et al., 2013, and Salthouse, 2012), older participants exhibited similar performances to younger participants in the use of common ground in the three studies included in this review (Horton & Spieler, 2007; Hupet et al., 1993; Lysander & Horton, 2012). Although there are few studies in this field, the results do not support the hypothesis that using common ground involves highly preserved executive control capacities.

The implication of theory of mind in the use of common ground also remains unclear given the divergent results from studies that investigated the ability to use common ground in patients with theory of mind difficulties. In their case study of a patient with bilateral amygdala damage, Gupta et al. (2011) showed that the decrease in the time taken to accomplish the task and in the number of words produced across trials was significantly lower for the patient compared to controls, while patients with vmPFC damage (Gupta et al., 2012) and adults with ASD (Nadig et al., 2015) did not differ from controls in their ability to build common ground with a given partner. Overall, there is no clear evidence that common ground is underpinned by executive functioning and theory of mind abilities despite theoretical arguments. Given the small number of clinical studies and given their methodological limitations (inclusion of a small number of patients, frequent use of an experimenter as a conversational partner, no statistical test for a link between common ground use and cognitive and social functions), further research in this field is needed.

### Limitations

It is important to acknowledge that this review presents a number of limitations. First, the inclusion criterion regarding the type of the conversational task used (i.e., paradigms that allow a spontaneous dialogue between partners who are explicitly encouraged to talk freely) may be questioned. As highlighted above, this decision was motivated by our desire to study both common ground construction *and* subsequent use by dialogue partners, but we acknowledge that one direct consequence of this decision was that some papers that only focused on one of these processes (e.g., common ground use only) were necessarily excluded from this work, despite their obvious interest for common ground research. Another limitation stems from our focus on linguistic measures of common ground, which led us to exclude studies that used non-linguistic measures of common ground (e.g., eye-tracking studies). Finally, some articles were excluded on the basis of the title and the abstract during the selection procedure (e.g., because they did not explicitly mention that they assessed the construction and the use of common ground using linguistic measures and/or they did not use the terminology related to the concept of common ground) when they in fact *did* involve such measures in experimental tasks corresponding to our inclusion criteria (e.g., Champagne-Lavau et al., 2009; Duff et al., 2013).^4^ Subsequent work is thus needed to determine how the conclusions drawn in the current paper may be generalized to other common ground markers and/or to different dialogue settings. Nonetheless, we believe that the present work represents an important first step towards understanding how common ground construction and use are examined in dialogue research.

## Conclusion

Using common ground during dialogue is an essential skill to ensure successful communication. It is therefore important to identify precise and reliable measures to assess common ground use in experimental dialogue settings. This review sheds light on the relevance of systematically using both direct and indirect linguistic measures while assessing common ground since these measures could reflect different concepts underpinned by distinct cognitive processes. Furthermore, the review highlights the lack of research addressing the question of social and cognitive mechanisms underlying common ground use and the need to develop more precise theoretical models that can account for the factors influencing the production and comprehension of utterances during dialogue. This should also help clinicians to apprehend more adequately the potential difficulties encountered by certain clinical populations in social interaction

## Footnotes

1 It is important to highlight that although participants may use their common ground from the second trial onwards in this kind of task, dialogue partners may also *update* their common ground in these trials (e.g., they might find a new way to refer to a picture that had already been referred to in previous trials). Thus, common ground construction may also occur in trials following the first trial. Nevertheless, an important feature of the first trial is that it constitutes the participants’ first joint encounter with the pictures. This is why we suggest that most of the participants’ common ground is built during the first trial in the matching task.

2 The keywords “language production” and “language comprehension” were both included because we were initially only interested in the distinction between linguistic and non-linguistic measures of common ground. However, because studies on language comprehension in dialogue tend to involve non-linguistic measures of common ground, and that we do not focus on such measures here, studies on dialogic comprehension are not included in this work, despite the inclusion of the keyword “language comprehension” in our initial search.

3 Note that this choice can be debated because the category includes very different elements (that cannot be dissociated here because they were used together within the same measure in some of the included studies), and it is therefore difficult to decide whether it is discourse or reference. We categorized it as discourse mainly because it includes the planning time before producing the first utterance.

4 We thank the reviewers for bringing to our attention these studies that indeed meet the inclusion and exclusion criteria but did not contain any of the keywords about common ground, and therefore did not appear in the database search.
